# Supplementary material for: Pyruvate Dehydrogenase Kinase 1 inhibition mediated oxidative phosphorylation enhancement in cartilage promotes osteoarthritis progression
Source: BMC Musculoskelet Disord. 2023 Jul 20;24:597. doi: 10.1186/s12891-023-06585-6 (PMC10357736; doi:10.1186/s12891-023-06585-6)
Supplement: Supplementary file 1 — Additional file 1. [file 12891_2023_6585_MOESM1_ESM.zip › BP.Gsea.1653623667859/GOBP_INOSITOL_PHOSPHATE_CATABOLIC_PROCESS.html]

Details for gene set GOBP\_INOSITOL\_PHOSPHATE\_CATABOLIC\_PROCESS[GSEA]

|  || Dataset | 55457\_collapsed\_to\_symbols.cls.cls #OA\_versus\_normal.cls.cls #OA\_versus\_normal\_repos |
| Phenotype | cls.cls#OA\_versus\_normal\_repos |
| Upregulated in class | normal |
| GeneSet | GOBP\_INOSITOL\_PHOSPHATE\_CATABOLIC\_PROCESS |
| Enrichment Score (ES) | -0.62834156 |
| Normalized Enrichment Score (NES) | -1.8686637 |
| Nominal p-value | 0.0017452007 |
| FDR q-value | 0.06267822 |
| FWER p-Value | 0.834 |
Table: GSEA Results Summary

  

Fig 1: Enrichment plot: GOBP\_INOSITOL\_PHOSPHATE\_CATABOLIC\_PROCESS      
 Profile of the Running ES Score & Positions of GeneSet Members on the Rank Ordered List

  

| SYMBOL | TITLE | RANK IN GENE LIST | RANK METRIC SCORE | RUNNING ES | CORE ENRICHMENT || 1 | NUDT3 | nudix hydrolase 3 [Source:HGNC Symbol;Acc:HGNC:8050] | 2870 | 0.264 | -0.1734 | No |
| 2 | MTMR7 | myotubularin related protein 7 [Source:HGNC Symbol;Acc:HGNC:7454] | 4432 | 0.119 | -0.2723 | No |
| 3 | INPP5J | inositol polyphosphate-5-phosphatase J [Source:HGNC Symbol;Acc:HGNC:8956] | 6009 | -0.005 | -0.3937 | No |
| 4 | BPNT2 | "3'(2'), 5'-bisphosphate nucleotidase 2 [Source:HGNC Symbol;Acc:HGNC:26019]" | 6136 | -0.015 | -0.4006 | No |
| 5 | MTMR2 | myotubularin related protein 2 [Source:HGNC Symbol;Acc:HGNC:7450] | 6189 | -0.019 | -0.4010 | No |
| 6 | INPP4B | inositol polyphosphate-4-phosphatase type II B [Source:HGNC Symbol;Acc:HGNC:6075] | 6284 | -0.026 | -0.4034 | No |
| 7 | SYNJ2 | synaptojanin 2 [Source:HGNC Symbol;Acc:HGNC:11504] | 6358 | -0.032 | -0.4030 | No |
| 8 | OCRL | OCRL inositol polyphosphate-5-phosphatase [Source:HGNC Symbol;Acc:HGNC:8108] | 7319 | -0.103 | -0.4582 | No |
| 9 | IMPA1 | inositol monophosphatase 1 [Source:HGNC Symbol;Acc:HGNC:6050] | 7332 | -0.105 | -0.4395 | No |
| 10 | INPP5K | inositol polyphosphate-5-phosphatase K [Source:HGNC Symbol;Acc:HGNC:33882] | 8103 | -0.163 | -0.4686 | No |
| 11 | INPP5A | inositol polyphosphate-5-phosphatase A [Source:HGNC Symbol;Acc:HGNC:6076] | 8813 | -0.218 | -0.4828 | No |
| 12 | INPP1 | inositol polyphosphate-1-phosphatase [Source:HGNC Symbol;Acc:HGNC:6071] | 10454 | -0.367 | -0.5413 | Yes |
| 13 | SYNJ1 | synaptojanin 1 [Source:HGNC Symbol;Acc:HGNC:11503] | 11577 | -0.512 | -0.5324 | Yes |
| 14 | IMPA2 | inositol monophosphatase 2 [Source:HGNC Symbol;Acc:HGNC:6051] | 11684 | -0.530 | -0.4413 | Yes |
| 15 | PTEN | phosphatase and tensin homolog [Source:HGNC Symbol;Acc:HGNC:9588] | 11836 | -0.556 | -0.3489 | Yes |
| 16 | INPP5E | inositol polyphosphate-5-phosphatase E [Source:HGNC Symbol;Acc:HGNC:21474] | 11882 | -0.568 | -0.2459 | Yes |
| 17 | NTSR1 | neurotensin receptor 1 [Source:HGNC Symbol;Acc:HGNC:8039] | 12677 | -0.849 | -0.1484 | Yes |
| 18 | INPP5B | inositol polyphosphate-5-phosphatase B [Source:HGNC Symbol;Acc:HGNC:6077] | 12723 | -0.884 | 0.0137 | Yes |
Table: GSEA details [plain text format]

  

Fig 2: GOBP\_INOSITOL\_PHOSPHATE\_CATABOLIC\_PROCESS      
 Blue-Pink O' Gram in the Space of the Analyzed GeneSet

  

Fig 3: GOBP\_INOSITOL\_PHOSPHATE\_CATABOLIC\_PROCESS: Random ES distribution      
 Gene set null distribution of ES for **GOBP\_INOSITOL\_PHOSPHATE\_CATABOLIC\_PROCESS**
